# Prevalence and risk factors of sarcopenia without obesity and sarcopenic obesity among Chinese community older people in suburban area of Shanghai: A cross-sectional study

**DOI:** 10.3389/fnagi.2022.1034542

**Published:** 2022-12-20

**Authors:** Linqian Lu, Xiangfeng He, Yanping Song, Min Zhuang, Xie Wu, Nan Chen

**Affiliations:** ^1^Department of Rehabilitation, Xinhua Hospital Affiliated to Shanghai Jiaotong University School of Medicine, Shanghai, China; ^2^Department of Rehabilitation, Xinhua Hospital Chongming Branch, Shanghai, China; ^3^School of Kinesiology, Shanghai University of Sport, Shanghai, China

**Keywords:** sarcopenia, sarcopenic obesity, prevalence, risk factor, older people, suburban

## Abstract

**Objectives:**

The aim of the present study was to explore the prevalence and risk factors of sarcopenia without obesity (S) and sarcopenic obesity (SO) among community-dwelling older people in the Chongming District of Shanghai, China, according to the Asian Working Group for Sarcopenia (AWGS) 2019 Consensus as the diagnostic criteria of sarcopenia.

**Methods:**

In this cross-sectional study, a total of 1,407 subjects aged ≥65 years were included, where the mean age of the subjects was 71.91 ± 5.59 years and their mean body mass index (BMI) was 24.65 ± 3.32 kg/m^2^. According to the Asian Working Group for Sarcopenia (AWGS) 2019 Consensus, sarcopenia was defined as a low appendicular skeletal muscle mass index (≤7.0 kg/m^2^ in males and ≤5.7 kg/m^2^ in females), decreased handgrip strength (<28.0 kg in males and <18.0 kg in females), and/or low gait speed (<1.0 m/s) or poor 5-time chair stand test (5CST) (≥12s). The SO met both the diagnostic criteria for sarcopenia and obesity, meanwhile obesity was defined as an increased percentage of body fat (PBF) (≥25% in males and ≥35% in females). Univariate and multiple logistic regression analyses were performed to explore the risk factors of both S and SO.

**Results:**

The prevalence of S and SO was 9.74% (M: 9.29%, F: 10.05%) and 9.95% (M: 13.94%, F: 7.14%). Lower BMI (OR = 0.136, 95% CI: 0.054–0.340, *p* < 0.001), lower hip circumference (OR = 0.858, 95% CI: 0.816–0.903, *p* < 0.001), farming (OR = 1.632, 95% CI: 1.053–2.530, *p* = 0.028), higher high-density lipoprotein cholesterol (HDL-C) level (OR = 2.235, 95% CI: 1.484–3.367, *p* < 0.001), and a sleep duration <7 h (OR = 0.561, 95% CI: 0.346–0.909, *p* = 0.019) were risk factors for S. While aging (70–74 y, OR = 1.923, 95% CI: 1.122–3.295, *p* = 0.017; 75–79 y, OR = 3.185, 95% CI: 1.816–5.585, *p* < 0.001; ≥80 y, OR = 7.192, 95% CI: 4.133–12.513, *p* < 0.001), male (OR = 1.981, 95% CI: 1.351–2.904, *p* < 0.001), higher BMI (OR = 4.865, 95% CI: 1.089–21.736, *p* = 0.038), higher monocyte level (OR = 4.203, 95% CI: 1.340–13.181, *p* = 0.014), and a sleep duration >9 h (OR = 1.881, 95% CI: 1.117–3.166, *p* = 0.017) were risk factors for SO.

**Conclusion:**

Our study showed the high prevalence of S and SO among community-dwelling older people in the Chongming District. The SO was more prevalent in males. Behavioral factors and lifestyle (such as farming and sleep duration) were associated more with the development of S, while age and male gender were associated more with the development of SO.

## 1. Introduction

Sarcopenia, a chronic and progressive disease associated with aging, is characterized by low skeletal muscle mass, decreased muscle strength, and/or reduced physical performance (Chen et al., 2020). It has several adverse outcomes such as falls, fractures, poor quality of life, and even premature mortality in older people (Tsekoura et al., 2017; Zhang et al., 2018, 2020). Obesity, as a prevalent disease among older population, is related to a variety of adverse outcomes, such as chronic low-grade inflammation state, metabolic diseases, cardiovascular disorders, and mortality (Calle et al., 2005; Ghoorah et al., 2016; Saltiel and Olefsky, 2017; Kawai et al., 2021). Sarcopenia and obesity are both associated with aging, which leads to a higher risk of having sarcopenia and obesity simultaneously in older people, resulting in a novel geriatric syndrome defined as sarcopenic obesity (SO) (Batsis and Villareal, 2018).

A number of studies have been conducted to explore the risk factors for total sarcopenia or SO alone. For example, a study by Chen et al. included age, body mass index (BMI), chronic diseases, physical activity (PA), and lifestyle as factors in the analysis to explore risk factors of sarcopenia, and found that only older age was an independent risk factor for sarcopenia (Chen et al., 2021). And a study by Wagenaar et al. included age, number of comorbidities, PA, education level, lifestyle, and nutrition intake as factors in the analysis to explore risk factors of SO, and found that only older age was a significant risk factor of SO (Wagenaar et al., 2021). However, most similar studies have not included comprehensive factors (such as blood biochemical indicators) in the analysis to explore risk factors for sarcopenia and SO, which may likely result in the analysis exploring these factors to be incomplete. It becomes explicit that there is a strong relationship between blood biochemical indicators and sarcopenia (Kalinkovich and Livshits, 2015). Skeletal muscle mass and fat mass are both closely related to lipoprotein cholesterol levels (Garrison et al., 1980; Vella et al., 2020). And white blood cell (WBC), as an immune cell type, is significantly associated with inflammatory diseases (such as sarcopenia) and reflects the severity of disease progression (Lee et al., 2019). Thus, it is necessary to identify whether the change in blood biochemical indicators' level is a risk factor for sarcopenia.

Currently, most studies focus on the prevalence and risk factors of total sarcopenia in urban areas (Hao et al., 2018; Kurose et al., 2020). However, a significant difference in the prevalence of total sarcopenia among older people between urban and suburban areas has been found, which shows that older people in suburban areas are more likely to suffer from sarcopenia than those in urban areas (Wu et al., 2021). Additionally, there are other differences between urban and suburban areas in terms of demographic characteristics (such as education level and occupational category) and lifestyles (such as physical activity [PA] level, sleep duration, and the quality of life) (Su et al., 2012; Zhang and Crimmins, 2019). Older people in suburban areas have a poorer quality of life and a lower subjective wellbeing compared with those in urban areas (Li et al., 2007). All the above differences are closely related to S or SO (Batsis and Villareal, 2018; Kitamura et al., 2021). However, a few large-scale studies have focused on the prevalence and risk factors of S and SO among community-dwelling older people in suburban areas.

Our study was performed in the Chongming District which is known to be one of the most aging areas in Shanghai, i.e., housing the maximum number of aging dwellers. So, it has a large population of older people. According to the 2021 population census data, the Chongming District had more than 0.18 million older people over 65 years, accounting for 29.6% of the total population of the entire Chongming District. The development of sarcopenia is significantly associated with increased age. A cross-sectional study by Therakomen et al. found that compared with the odds ratio of older people aged 60–69 years, the odds ratio (OR) of sarcopenia in those aged 70–79 years was 6.87 and in those aged ≥80 years it was 13.71 (Therakomen et al., 2020). Thus, it is necessary to explore the prevalence and risk factors of S and SO in the Chongming District, whose population is seriously aging. Additionally, the Chongming District is a typical suburban area located on a separate island, and its whole area is independent of the urban area of Shanghai. This special geographical location leads to low population mobility, especially for older people, and most of them are mainly engaged in farming. All the above characteristics affect the developmental activity in the fields of both economy and medicine. As a result, the awareness of S and SO is lacking among medical staff and older people. So, further studies on S and SO should be performed to raise the awareness of S and SO among the population in this suburban area.

Thus, this study aims to conduct a large-scale study to investigate and explore the prevalence and risk factors of S and SO among community-dwelling older people in the Chongming District, Shanghai, to improve the attention of older people to S and SO, and to provide clinical suggestions for the prevention and treatment of S and SO.

## 2. Materials and methods

### 2.1. Study design

In this cross-sectional study, the data on older people living in the Chongming District were collected from April to December in the year 2021 at the Chongming Chengqiaozhen Community Health Service Center in the Chongming District of Shanghai, China. The comprehensive assessments included a number of clinical characteristics (including age, gender, anthropometrics, demographics, and chronic diseases), cardiovascular disease (CVD) risk factors (including inflammation factors and blood lipid factors), PA level, and lifestyle habits of the elderly population.

### 2.2. Study subjects

Our study was performed in the Chongming District, a typical suburban area located on an island independent of the urban area of Shanghai. And we recruited subjects from two towns in the Chongming District (Chengqiao town and Sanxing town), and there were five communities in Chengqiao town (*n* = 5,286) and two communities in Sanxing town (*n* = 2,035). A total of seven communities have similar demographic characteristics and health statuses. All residents ≥65 years in seven communities (*n* = 7,321) were invited to participate in our study, and eventually, a total of 1,558 community-dwelling older people were willing to participate in our study. Before the assessments, informed consent form was filled in, duly signed, and handed over by each subject. Due to personal reasons or inability to complete the assignment within the timeframe, a total of 1,407 community-dwelling older people (aged 71.91 ± 5.59 years) were finally included in our study. The eligible subjects were included, if: (1) age ≥ 65 years; (2) living in the community for a long time independently (living in their own house or living together with family); and (3) willing to participate in our study and take part in various assessments. Subjects who met the following criteria were not included in the study: (1) have any physical disabilities or dysfunctions in terms of cognitive, hearing, or visual impairment which would affect the study process and (2) cannot communicate with researchers during the process of assessments.

### 2.3. Definitions of sarcopenia and sarcopenic obesity

Our study chose the Asian Working Group for Sarcopenia (AWGS) 2019 Consensus as the diagnostic criteria of sarcopenia (Chen et al., 2020). Low appendicular skeletal muscle mass index (ASMI) was measured by the bioelectrical impedance analysis (BIA) (Inbody 720, Korea), with cut-off values of appendicular skeletal muscle mass ≤7.0 kg/m^2^ in males and ≤5.7 kg/m^2^ in females. The decreased handgrip strength (HGS) was measured by the hand dynamometer (Jamar Plus+ Digital Hand Dynamometer, IL, USA), with cut-off values of decreased handgrip strength <28.0 kg in males and <18.0 kg in females. And physical performance was measured by the 6-meter walking test or the 5-time chair stand test (5CST), and the cut-off values of low physical performance were <1.0 m/s on gait speed (GS) or ≥12.0 s for the time of 5CST.

The diagnostic criteria for SO should meet the criteria of both sarcopenia and obesity, simultaneously. For the diagnosis of obesity, we chose the method recommended by the World Health Organization (WHO), with the cut-off values as the percentage of body fat (PBF) ≥25% in males and PBF ≥35% in females (World Health Organization, 1995, 2000).

### 2.4. Assessments

Each subject performed each assessment in turn following a standardized procedure for all assessments that were later measured by qualified professional researchers. And all subjects' parameters were measured in the appropriate physical condition. If the subjects became physically fatigued due to any recent work, they were advised to take rest for several days before taking up the assessment once again. This guideline had to be strictly followed to avoid bringing down the accuracy of the assessments due to subject's acute work fatigue.

#### 2.4.1. Assessment of anthropometric characteristics

The assessment of anthropometric characteristics included the height, weight, calf circumference (CC), waist circumference (WC), and hip circumference (HC) of the subject. It is important to ensure that the subjects wear tight clothes and avoid wearing thick clothes during the measurement process, so as not to affect the measurement results. The body mass index (BMI) was calculated in our study.

#### 2.4.2. Assessment of body composition

Body composition of the subject was measured with BIA by trained researchers, following a standardized protocol that had been validated; meanwhile, the subjects were positioned for assessment, as required by the manufacturer's guidelines. Before using the BIA, the researchers calibrated the machine according to the manufacturer's guideline to ensure the accuracy of the measurement results each time. The assessments of body composition included appendicular skeletal muscle mass (ASM), which was calculated as the sum of the skeletal muscle mass of the upper and the lower limbs, body fat mass, and PBF. Besides, ASMI was calculated by the formula ASMI = ASM (kg)/height^2^ (m), which was recommended by the AWGS 2019 Consensus.

#### 2.4.3. Assessment of demographic characteristics

Demographic characteristics of the subjects were recorded. The subjects who were engaged in activities, such as pulling grass, fishing, shepherding, and farming, were defined as farmers. The education level of the subjects was divided into three categories, of which lower education level included subjects who were not educated and subjects who received only primary education, moderate education level included junior high school education and senior high school education, and high education level included undergraduate education and above.

Smoking and drinking status of the subjects were known by asking the subjects face to face, and both were in turn divided into three categories including never, current, and former smoking/drinking. Nutrition status was measured by trained researchers who conducted the Mini Nutritional Assessment, which is a simple and high-sensitive nutritional tool for screening to assess the nutritional condition of subjects (Cereda, 2012). The Mini Nutritional Assessment included the following four parts: anthropometric assessments (such as body height, body weight, and CC), global evaluations (such as lifestyle, type of residence, drug use, and mental/psychological condition), dietetic evaluations (such as a number of meals per day, appetite situation, type of food intake, and way of food intake), and subjective assessments (such as self-awareness of health and nutritional status). Based on the subjects' answers, a certain score was assigned to each item of the four parts (a total of 24 items), and the total score ranged from 0 to 30 points. The total score of <17.0 points indicated that the subjects were suffering from protein-calorie malnutrition, 17.0–23.5 points indicated that the subjects were at risk of malnutrition, and ≥24.0 points indicated that the subjects had adequate nutritional status.

The depression status was measured by the Geriatric Depression Scale-15, which is a tool for the diagnosis and evaluation of depression status in older people (Tang, 2013). In this questionnaire, a total of 15 items were included, and the total score ranged from 0 to 15 points, and the higher score, the more obvious symptoms of depression. In our study, a score of <5 points was scored as no depressive symptoms, and ≥5 points was scored as depressive symptoms (Low et al., 2020).

#### 2.4.4. Assessment of chronic diseases

Hypertension was defined by the following criteria: (1) has been diagnosed by a professional clinician; (2) or is currently taking antihypertensive medication; (3) or the systolic blood pressure of subjects ≥140 mmHg and/or diastolic blood pressure ≥90 mmHg at the baseline assessment (NCD Risk Factor Collaboration, 2021). The blood pressure was measured using an automated blood pressure device (Omron HEM-907, Tokyo, Japan). Subjects were defined as having dyslipidemia, if they met the following criteria (Joint Committee on Revision of Guidelines for Prevention Treatment of Dyslipidemia in Chinese Adults, 2016): (1) have been diagnosed by a professional clinician; (2) or are currently taking lipid-lowering drugs; (3) or the total cholesterol (TCHOL) ≥6.2mmol/L; or triglycerides (TG) ≥2.3 mmol/L; or low-density lipoprotein cholesterol (LDL-C) ≥4.1 mmol/L; or high-density lipoprotein cholesterol (HDL-C) <1.0 mmol/L. Those who met the following criteria were diagnosed with type 2 diabetes: (1) have been diagnosed by a professional clinician; (2) or are currently taking hypoglycemic drugs; (3) or a fasting plasma glucose ≥7.0 mmol/L at the baseline assessment (Kuzuya et al., 2002). If the subject has diseases of the heart, such as coronary heart disease, ischemic heart diseases (such as angina pectoris, myocardial ischemia, and myocardial infarction), and cardiac arrhythmia that can be defined as having heart disease. If the subject has a history of ischemic or hemorrhagic stroke, it can be defined as having a stroke.

#### 2.4.5. Assessment of cardiovascular disease risk factors

For each subject, the fasting venous blood sample after a 12-h overnight fasting was collected by a professional nurse in the morning at about 08:00 am−09:00 am. Inflammation factors (including WBC, blood platelets, lymphocytes, monocytes, neutrophils, and eosinophils) were included in our study. And the ratio of platelets to lymphocytes, that of lymphocytes to monocytes (LMR), and that of neutrophils to lymphocytes were also calculated in our study. In addition, blood lipid factors (including TCHOL, TG, LDL-C, and HDL-C) were also recorded and analyzed.

#### 2.4.6. Assessment of physical activity level and lifestyle

The short form International Physical Activity Questionnaire (IPAQ-SF) in Chinese language was conducted to assess the PA level of subjects (Qu and Li, 2004). The IPAQ-SF consists of three PA intensities (including walking, moderate-intensity, and vigorous-intensity) and sitting. And the frequency (days/week) and duration (min/day) of three intensity PAs in the past seven days were also recorded. The time spent in each intensity PA per day (min/day) was multiplied by the number of days per week (days/week) in which the subject participated, then multiplied by the metabolic equivalent tasks (METs) of the corresponding PA intensity, and the final value is the PA level per week. The MET value for walking is 3.3, meanwhile for moderate-intensity PA it is 4 and for vigorous-intensity PA it is 8. The total weekly PA level, which is determined by the sum of three PA levels per week (including walking, moderate-intensity, and vigorous-intensity), is classified into low, moderate, or high PA level.

Low PA level can be defined, if it met the following criteria: (1) No reported activity; or (2) reported a part of activities, but did not meet the requirements for moderate or high PA level. Moderate PA level can be defined, if it met the following criteria: (1) At least 20 min of all types of vigorous-intensity PA per day, for a total number of days ≥3 days; or (2) at least 30 min of all types of moderate-intensity and/or walking per day, for a total number of days ≥5 days; or (3) total number of days of three PA intensities ≥5 days and a total weekly PA level of ≥600 MET-min/week. High PA level can be defined, if it met the following criteria: (1) a total number of days of all types of vigorous-intensity PA ≥3 days and a total weekly PA level of ≥1,500 MET-min/week; (2) a total number of days of three PA intensities ≥7 days and a total weekly PA level of ≥3,000 MET-min/week.

A lifestyle PA questionnaire was used to assess the PA of lifestyle. The assessments include whether the subjects have exercise habits per day, self-reported sitting time, housework time, and sleep duration per day. Types of exercise include regular aerobic exercise, running, swimming, or cycling. Sitting time refers to the time subjects spend in watching television or looking at electronic gadgets such as mobile phones. Housework time refers to the time when the subjects sweep the floor, mop the floor, cook, tidy up the room, and so on. The daily sleep duration of subjects is divided into three levels as <7 h, 7–9 h, and >9 h, and each subject was asked the time when they would go to sleep and when they would get up the next day in the previous seven days. And the mean daily sleep duration was calculated.

### 2.5. Statistical analysis

All statistical analyses were conducted by the IBM SPSS Statistics version 24.0 (SPSS, Inc., USA). All data were presented as numbers, mean ± standard deviation, and percentages. Before analyzing the continuous variables, we performed the Common Normality Tests and used the independent samples *t-*tests for normally distributed continuous data, meanwhile the Mann–Whitney *U* test was used for non-normally distributed continuous data.

To analyze the differences between the non-sarcopenic obesity (NSO) group (including normal, S, and obesity without sarcopenia [O] group) and the SO group, our study selected a one-way analysis of variance (ANOVA) test for analyzing continuous variable data (using Bonferroni, Tukey, and Games-Howell correction of adjustment), while the Pearson's chi-square test was used for the comparison of categorical variable data (using Bonferroni's correction of adjustment). Univariate and multiple logistic regression analyses were performed to explore the associated risk factors of S and SO in older people, and OR and 95% confidence intervals (95% CIs) were recorded. The regression analysis was performed with the forward selection: likelihood ratio (LR) method as the variable screening method. The variables with *p-*value < 0.10 in the univariate logistic regression analysis would be included in multiple logistic regression analysis. For all data statistics, the *p-*value < 0.05 is considered to be statistically significant.

## 3. Results

### 3.1. Baseline characteristics

**Figure 1:**
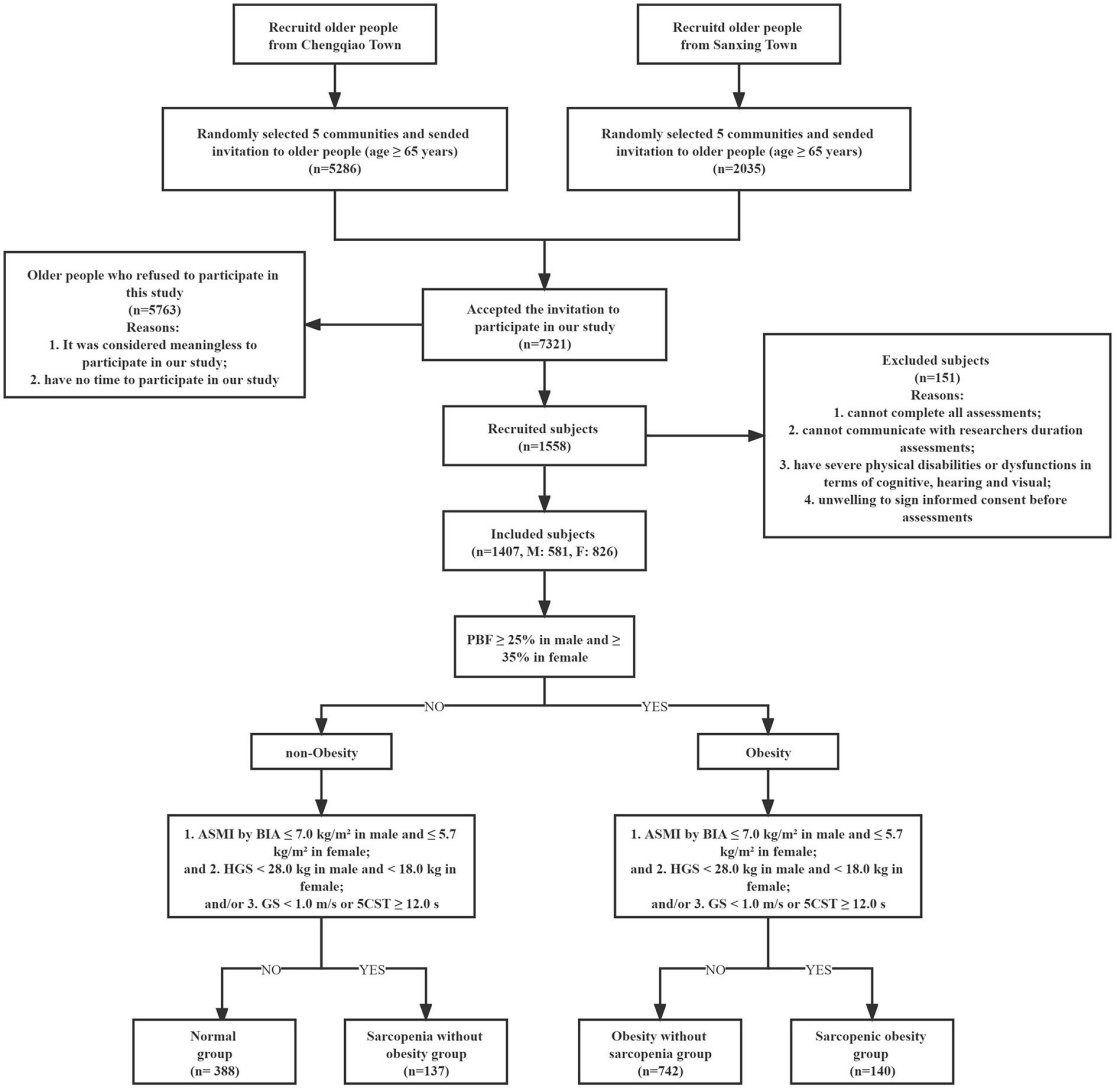
The flowchart of our study.

As many as 1,407 subjects aged ≥ 65 years (M: 581, F: 826) were finally included according to the AWGS 2019 Consensus in this cross-sectional study. Figure 1 shows the flowchart of our study. Among included subjects, the mean age was 71.91 ± 5.59 years old, mean BMI was 24.65 ± 3.32 kg/m^2^, mean height was 1.60 ± 0.08 m, and mean weight was 63.47 ± 10.63 kg. The details of baseline characteristics of included subjects are shown in Table 1.

**Table 1:** Baseline characteristics of included subjects.

**Variables**	**Total**	**Normal**	**Sarcopenia without obesity**	**Obesity without sarcopenia**	**Sarcopenic obesity**
Population, *n* (%)	1,407 (100)	388 (27.576)	137 (9.737)	742 (52.736)	140 (9.950)
Age (y), mean ± SD	71.91 (5.589)	70.25 (4.734)	73.53 (6.501)	71.70 (5.140)	76.06 (6.592)
BMI (kg/m^2^), mean ± SD	24.645 (3.316)	22.999 (2.471)	19.930 (1.704)	26.633 (2.508)	23.282 (2.325)
Height (m), mean ± SD	1.602 (0.077)	1.603 (0.073)	1.581 (0.083)	1.610 (0.076)	1.581 (0.083)
Weight (kg), mean ± SD	63.467 (10.627)	59.219 (8.295)	49.917 (6.256)	69.170 (8.865)	58.274 (7.840)
CC (cm), mean ± SD	34.011 (3.038)	33.411 (2.318)	30.404 (2.146)	35.349 (2.580)	32.121 (3.379)
SBP (mmHg), mean ± SD	148.67 (19.201)	147.63 (18.632)	143.72 (20.124)	149.87 (19.016)	150.02 (20.054)
DBP (mmHg), mean ± SD	79.43 (10.681)	78.90 (10.437)	76.10 (11.125)	80.40 (10.279)	78.98 (12.223)
HR (time/min), mean ± SD	74.87 (5.926)	74.65 (5.453)	74.18 (6.700)	75.11 (5.947)	74.91 (6.250)
ASMI (kg/m^2^), mean ± SD	6.792 (1.038)	6.738 (0.793)	5.723 (0.722)	7.176 (1.022)	5.953 (0.669)
HGS (kg), mean ± SD	28.010 (9.874)	27.166 (9.196)	24.285 (8.961)	29.617 (10.108)	25.484 (9.576)
GS (m/s), mean ± SD	0.835 (0.190)	0.881 (0.180)	0.771 (0.182)	0.846 (0.184)	0.705 (0.186)
5CST (s), mean ± SD	9.535 (3.492)	8.845 (2.394)	9.892 (3.394)	9.384 (3.360)	11.900 (5.362)
**History of falls**, ***n*** **(%)**					
Never	1,174 (83.4)	332 (85.6)	108 (78.8)	622 (83.8)	112 (80.0)
1 time	141 (10.0)	32 (8.2)	15 (10.9)	78 (10.5)	16 (11.4)
≥2 times	92 (6.5)	24 (6.2)	14 (10.2)	42 (5.7)	12 (8.6)
**Monthly income**, ***n*** **(%)**					
<2,000 RMB	497 (35.3)	131 (33.8)	51 (37.2)	255 (34.4)	60 (35.3)
2,000–5,000 RMB	775 (55.1)	230 (59.3)	75 (54.7)	405 (54.6)	65 (46.4)
5,001–10,000 RMB	(7.4)	20 (5.2)	7 (5.1)	65 (8.8)	12 (8.6)
>10,000 RMB	31 (2.2)	7 (1.8)	4 (2.9)	17 (2.3)	3 (2.1)

BMI, body mass index; CC, calf circumference; SBP, systolic blood pressure; DBP, diastolic blood pressure; HR, heart rate; ASMI, appendicular skeletal muscle mass index; HGS, handgrip strength; GS, gait speed; 5CST, 5-time chair stand test.

### 3.2. The prevalence of sarcopenia without obesity and sarcopenic obesity

**Figure 2:**
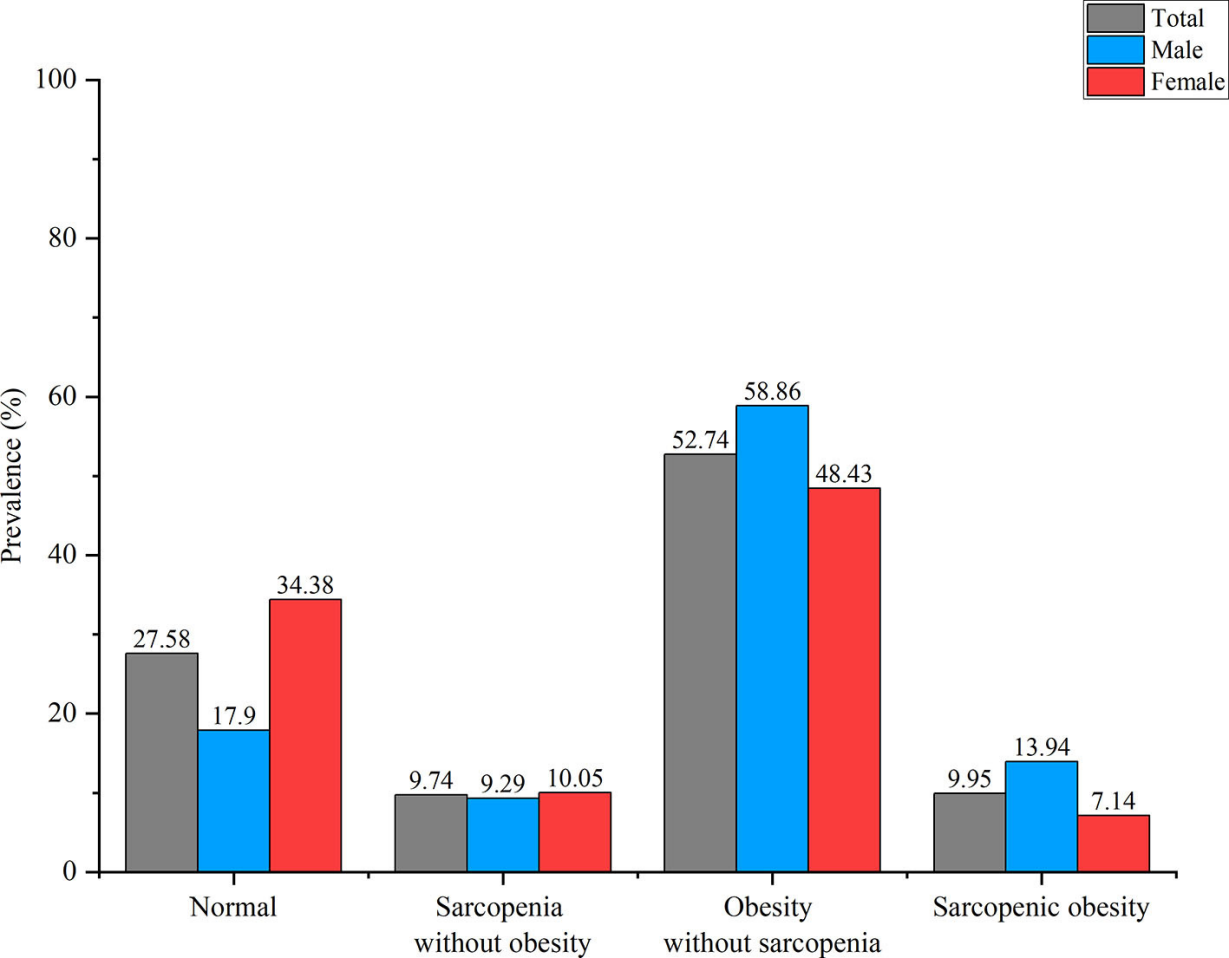
The prevalence of sarcopenia without obesity (S) and sarcopenic obesity (SO) stratified by gender.

**Figure 3:**
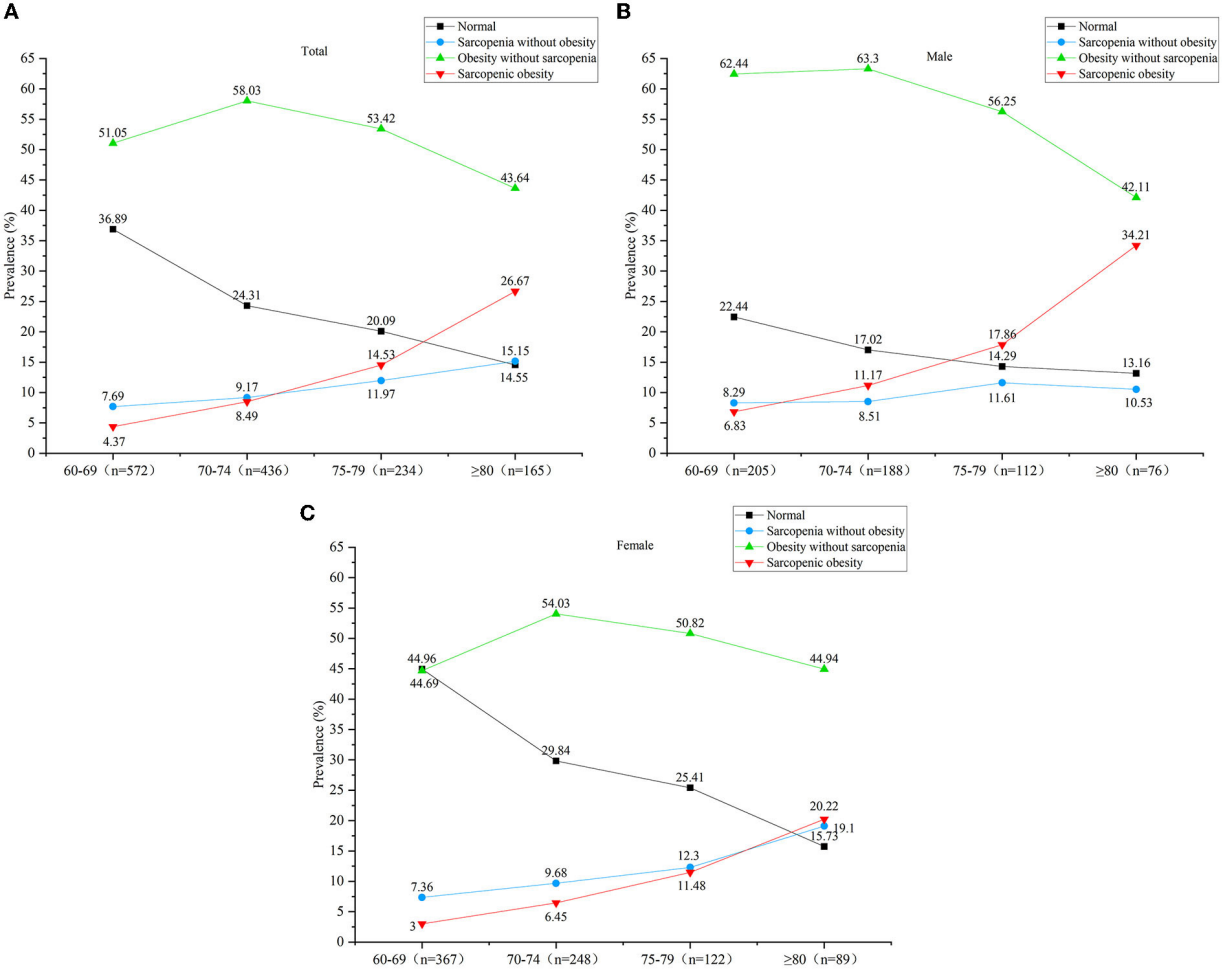
**(A)** The trend in the prevalence of sarcopenia without obesity (S) and sarcopenic obesity (SO) stratified by age in all subjects. **(B)** The trend in the prevalence of sarcopenia without obesity (S) and sarcopenic obesity (SO) stratified by age in males. **(C)** The trend in the prevalence of sarcopenia without obesity (S) and sarcopenic obesity (SO) stratified by age in males.

According to the diagnostic criteria for sarcopenia and obesity, 388 subjects (M: 104, F: 284) who had no sarcopenia or obesity were classified into the normal group, 137 subjects (M: 54, F: 83) were classified into the S group, 742 subjects (M: 342, F: 400) were classified into the O group, and 140 subjects (M: 81, F: 59) were classified into the SO group. Figure 2 shows the prevalence of S and SO stratified by gender. With aging, the prevalence of S and SO increased gradually both in males and females. Figures 3A–C shows the trend in the prevalence of S and SO stratified by age and gender.

### 3.3. The prevalence of sarcopenia without obesity and sarcopenic obesity by tertiles of physical activity level

**Figure 4:**
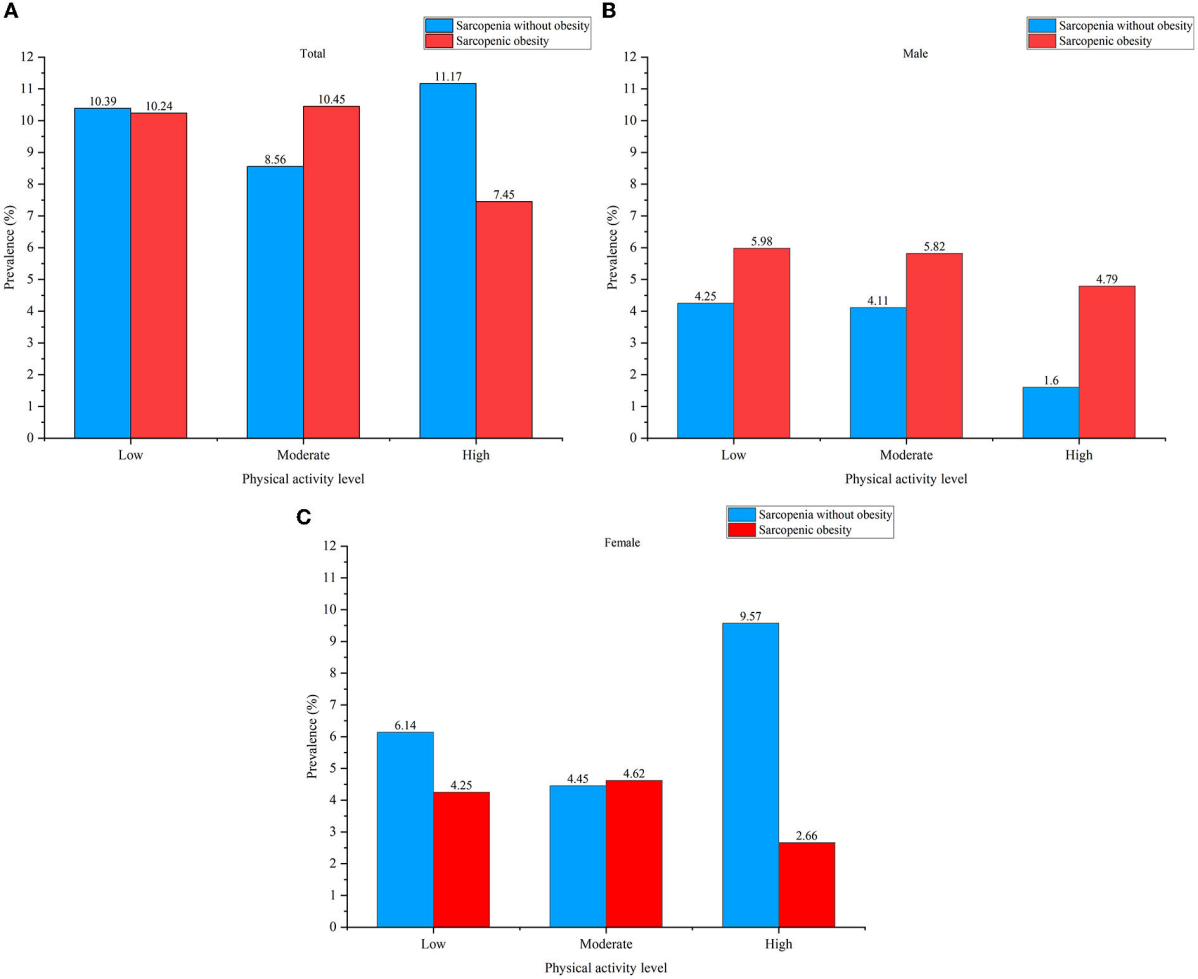
**(A)** The prevalence of sarcopenia without obesity (S) and sarcopenic obesity (SO) in low, moderate, and high physical activity (PA) levels in all subjects. **(B)** The prevalence of sarcopenia without obesity (S) and sarcopenic obesity (SO) in low, moderate, and high physical activity (PA) levels in males. **(C)** The prevalence of sarcopenia without obesity (S) and sarcopenic obesity (SO) in low, moderate, and high physical activity PA levels in all females.

In the low PA group, the total prevalence of S and SO was 10.39 and 10.24%, while in moderate and high PA groups the total prevalence was 8.56 and 10.45%, 11.17 and 7.45%, respectively. Figures 4A–C shows the prevalence of S and SO in low, moderate, and high PA levels.

### 3.4. Clinical characteristics and differences between the non-sarcopenic obesity group and the sarcopenic obesity group

There are significant differences between the S group and the SO group in terms of age, gender, BMI, WC, HC, PBF, nutritional status, and number of comorbidities (Supplementary Table 1a). Compared with the S group, the SO group has an older mean age (76.06 ± 6.59 y vs. 73.53 ± 6.50 y, *p* < 0.001), and the proportion of subjects in the SO group aged 65–69 years was significantly lower than the proportion of subjects in the SO group (17.9 vs. 32.1%, *p* < 0.05). As for gender, the SO group has more males (57.9 vs. 39.4%) and fewer females (42.1 vs. 60.6%) than the S group (*p* < 0.05). In addition, there are significant differences between the S subjects and the SO subjects in terms of monocytes and HDL-C (*p* < 0.05 and *P* < 0.001) (Supplementary Table 1b). Supplementary Table 1c shows that there are significant differences between the NSO subjects and the SO subjects in terms of housework time and sleep duration (*p* = 0.001 and *p* = 0.010).

### 3.5. The risk factors of sarcopenia without obesity and sarcopenic obesity

**Figure 5:**
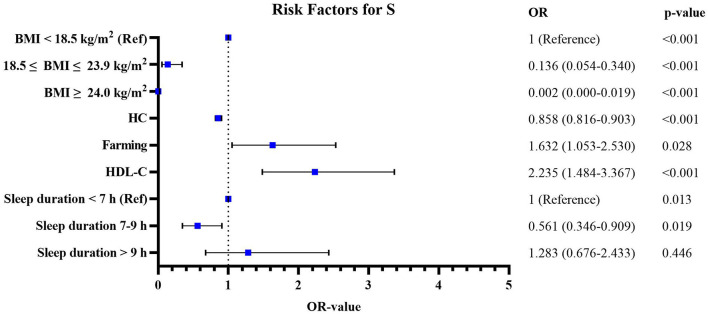
The risk factors for sarcopenia without obesity (S) according to the univariate and multivariate logistic regression analyses.

**Figure 6:**
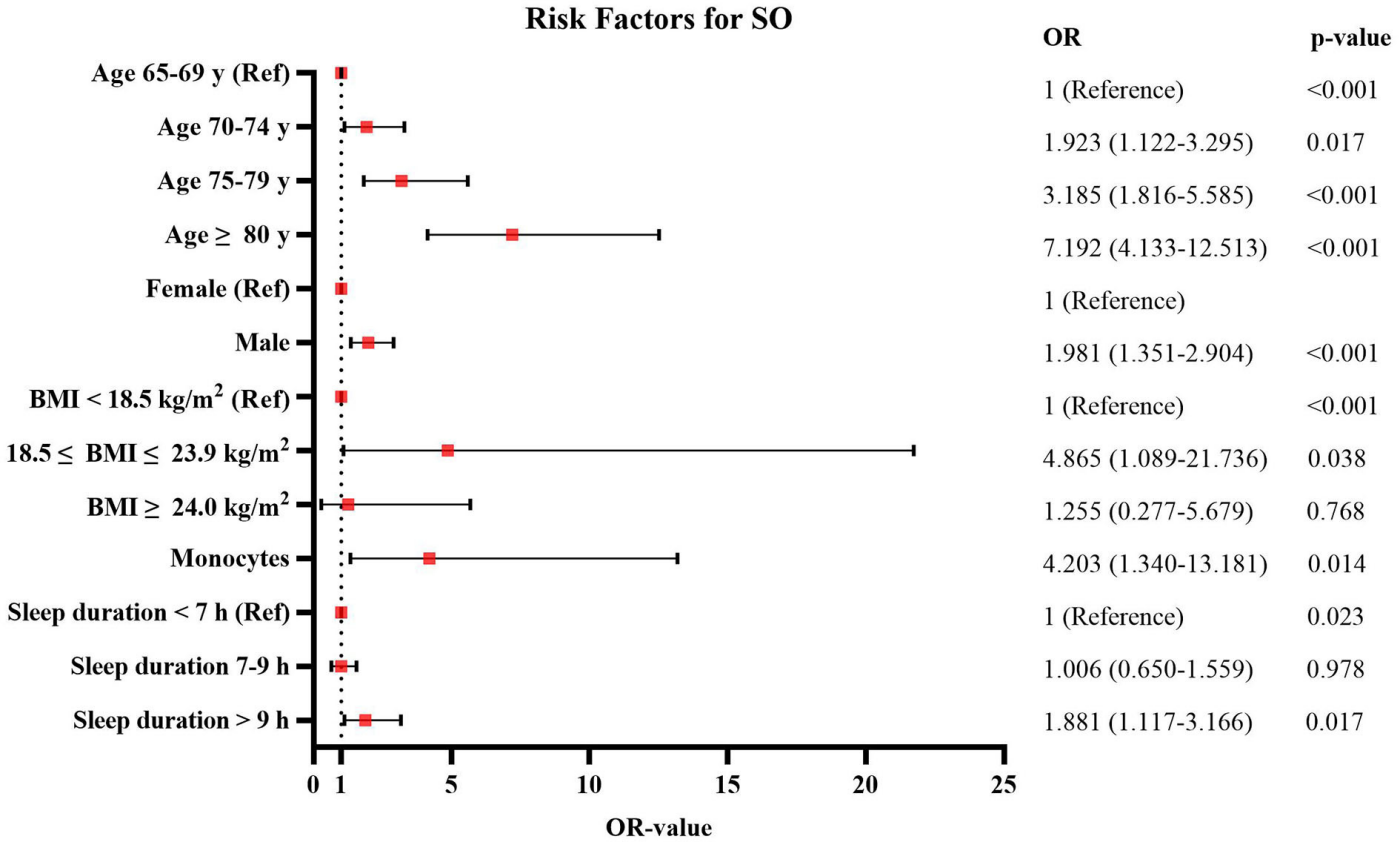
The risk factors for sarcopenic obesity (SO) according to the univariate and multivariate logistic regression analyses.

Figures 5, 6 shows the risk factors for S and SO in our study. Compared with the subjects aged 65–69 years, the odds ratio of SO among subjects aged 70–74 years was 1.923 (95% CI: 1.122–3.295, *p* = 0.017), aged 75–79 years it was 3.185 (95% CI: 1.816–5.585, *p* < 0.001), and aged ≥80 years it was 7.192 (95% CI: 4.133–12.513, *p* < 0.001). Compared with females, the odds ratio of SO among males was 1.981 (95% CI: 1.351–2.904, *p* < 0.001). Compared with those subjects not working on farming, the odds ratio of S among working on farming subjects was 1.632 (95% CI: 1.053–2.530, *p* = 0.028), while farming was not a risk factor for SO. An increase in monocytes was positively associated with an increased risk of SO (OR = 4.203, 95% CI: 1.340–13.181, *p* = 0.014). And with the increasing level of HDL-C, the risk of S showed a significant upward trend (OR = 2.235, 95% CI: 1.484–3.367, *p* < 0.001). Supplementary Table 2 shows the complete results of univariate and multiple logistic regression analyses of the risk factors for S and SO.

## 4. Discussion

In our study, S and SO were found to have high prevalence rates of 9.74 and 9.95%, respectively. Additionally, our study explored risk factors in groups of subjects with S and SO, and found that lower BMI, lower HC, farming, higher HDL-C level, and a sleep duration <7 h were risk factors for S, meanwhile aging, male gender, higher BMI, higher monocyte level, and a sleep duration >9 h were risk factors for SO. To the best of our knowledge, this study is the first cross-sectional study to investigate and explore the prevalence and associated risk factors of S and SO among community-dwelling older people in the Chongming District of Shanghai, China.

The prevalence of S was 9.74% in our study, and in a comparison of studies among Chinese population, we found that the prevalence of S was significantly different from those of previous studies (Du et al., 2019; Pasdar et al., 2021). A study by Du et al. demonstrated that the prevalence of S was 8.24% in the Chongming District, which is an urban area in Shanghai, China (Du et al., 2019), and which was lower than the result of our study. We speculated that it was due to the differences in population and regional characteristics between urban and suburban areas. Our study was conducted in the Chongming District, a suburban area of Shanghai, China, which is mainly inhabited by middle-aged and older people who are engaged in farming. On the contrary, the subjects included in Du et al. (2019) study did not engage in farming. A study by Lee et al. proved that the occupational category was associated with muscle strength that manual workers who were skilled in agriculture, forestry, fishery, or related trades workers had a higher risk of weak HGS than those non-manual workers (Lee, 2021). The repetitive movements and heavy works in agricultural activities reduce joint movement and inhibit skeletal muscle reflexes, which can damage the skeletal muscle function and result in muscle weakness (Russo et al., 2006). Under such intensive agricultural activities, the risk of musculoskeletal disorders such as sarcopenia may increase. And our study showed that engaging in farming was a risk factor for S (OR = 1.632, 95% CI: 1.053–2.530), and it may be the reason for making the prevalence of S in our study to be higher than Du et al. (2019) study. Therefore, we recommend that older people engaged in farming should pay attention to the posture and duration of work to avoid muscle fatigue and even muscle weakness. Additionally, region characteristic was also closely associated with sarcopenia. The economic, educational, and medical levels of the Chongming District are lower than those of the urban area of Shanghai, which may lead to a lack of self-awareness of health and nutritional management among older people in this area. Our study also found that over 90% of older people in the Chongming District were at risk of malnutrition. We speculated that the reason for malnutrition among older people may be due to an inappropriate dietary structure (such as lacking adequate protein intake). Several studies proved that low protein intake increased the risk of sarcopenia (Lin et al., 2021; Rogeri et al., 2021). Therefore, future studies should focus on the relationship between the dietary structure and sarcopenia among older people in the Chongming District, recommend a suitable diet for them, and prevent them from suffering from sarcopenia.

The prevalence of SO was 9.95% in our study, which was significantly higher than those of previous studies (Kemmler et al., 2016; Wagenaar et al., 2021). A cohort study by Wagenaar et al. demonstrated that the prevalence of SO was 1.19% by using 24 h urinary creatinine excretion to diagnose sarcopenia and using BMI to diagnose obesity (Wagenaar et al., 2021). Similarly, a study by Kemmler et al. demonstrated that the prevalence of SO was 2.3% by using ASMI, HGS, and GS to diagnose sarcopenia, meanwhile they used BMI to diagnose obesity (Kemmler et al., 2016). We speculated that the difference in the prevalence of SO was due to the different diagnostic criteria of sarcopenia and obesity. Wagenaar et al. (2021) study used the 24 h urinary creatinine excretion to diagnose sarcopenia. Due to the creatinine being formed from the components of muscle (creatinine and creatine phosphate) through the non-enzymatic conversion, its amount depends on the amount of muscle mass (Stam et al., 2019). In a steady state, creatinine is produced at a constant rate, while creatinine excretion can be affected under the effect of external factors (such as protein intake and kidney function) (Naser et al., 2020), which influences the diagnosis of sarcopenia further. Thus, using 24 h urinary creatinine excretion to diagnose sarcopenia is lower than using an anthropometric indicator such as muscle mass. In addition, our study used the PBF to diagnose obesity, while Wagenaar et al. (2021) and Kemmler et al. (2016) studies used the BMI to diagnose obesity. With aging, older people experience a decrease in muscle mass and an increase in fat mass, which lead to less significant changes in the total body weight (Zamboni et al., 2005; Wannamethee and Atkins, 2015). BMI does not reflect changes in body composition well that cannot be a sensitive indicator to identify obesity. Thus, using PBF may be more suitable to identify obesity than using BMI.

Our study showed that the prevalence of S and SO both gradually increased with aging. Several studies have also proved that the prevalence of S and SO both increased with aging among community-dwelling older people (Alexandre et al., 2014; De Campos et al., 2020). With increasing age, the prevalence of SO showed a rapidly increasing trend in our study, that compared with subjects aged 65–69 years, subjects aged 70–74 years have a 1.923-fold risk of SO, in aged 75–79 years have a 3.185-fold risk of SO, and in age ≥80 years old have a 7.192-fold risk of SO. We speculated that it is due to the change in adipose tissue, which played a substantial role in the development of SO. With aging, the accumulation of pro-inflammatory factors and various adipokines occurs in adipose tissue, then these adipose tissues ectopically accumulate in the skeletal muscle, resulting in muscle dysfunction. In turn, the inflammatory state of adipose tissue may be exacerbated and worsen the development of SO (Kalinkovich and Livshits, 2017).

We also found that males have a significantly higher risk for SO than females (OR = 1.981, 95% CI: 1.351–2.904). These results were not consistent with previous studies, which were mostly conducted in urban areas (Wagenaar et al., 2021; Yin et al., 2021). It may be due to the difference in PA levels between males and females in the Chongming District. Compared with low and moderate PA levels, a high PA level was associated with a lower prevalence of SO in males in our study. Compared with older females, older males engage in more intensive agricultural activities such as shoveling and weeding. These intensive agricultural activities involve long-term and repeated joint movements (such as bending neck, squatting, and kneeling) that can easily lead to muscle fatigue in older people (Kang et al., 2021). Long-time agricultural activities make older people have no enough relaxation time for muscle recovery, which leads to musculoskeletal disorders (Kang et al., 2021) and avoidance of PAs. The decreased level of PA may lead to an increase in fat mass, and it has been proved that inadequate PA was associated with the risk of SO (Mendham et al., 2021; Wagenaar et al., 2021). Therefore, we recommend that older people (especially males) should increase PA time and maintain an appropriate PA level, and control the time spent in agricultural activities, to prevent the development of sarcopenia.

Additionally, our study found that a sleep duration <7 h (OR = 0.561, 95% CI: 0.346–0.909) was also a risk factor for S. Several studies have also demonstrated that inadequate sleep duration was associated with an increasing risk of S (Chien et al., 2015; Hu et al., 2017). We speculated that it was due to the effect of inadequate sleep duration on endocrine function (Lucassen et al., 2012). In the state of inadequate sleep duration, the levels of hormones and pro-inflammatory factors show a declining trend [such as testosterone and insulin-like growth factor 1 (IGF-1)] (Chennaoui et al., 2020; Su et al., 2021), and the synthesis of muscle protein gets weakened, thereby affecting the skeletal muscle mass and developing into sarcopenia. In addition, a sleep duration of >9 h per day (OR = 1.881, 95% CI: 1.117–3.166) was a significant risk factor for SO in our study. This result was consistent with that of Kwon et al.'s study (Kwon et al., 2017). In Kwon et al. (2017)'s study, excessive sleep duration (≥9 h) was significantly more likely to have sarcopenia (including S and SO) among the Korean older people. It has been proven that excessive sleep duration can lead to the disruption of a body's circadian rhythm and the development of a chronic inflammatory state, and these body changes can reduce muscle protein synthesis and even lead to muscle proteolysis (Cesari et al., 2012; Piovezan et al., 2015). Therefore, inadequate or excessive sleep duration can increase the risk of S and SO, and a proper sleep duration is most beneficial for skeletal muscle synthesis.

In our study, we found that higher HDL-C level was a risk factor for S in older people. This result was consistent with those of previous studies (Habib et al., 2020; Liu et al., 2020; Wang et al., 2020). HDL-C was associated with glucose uptake in skeletal muscle cells (Drew et al., 2009; Lehti et al., 2013). The increasing HDL-C decreases insulin resistance levels (Andrew et al., 2010), leading to an increase in circulating glucose levels and a decrease in muscular glucose uptake, resulting in muscle dysfunction and eventually turning into sarcopenia (Merz and Thurmond, 2020). Monocytes, as a subtype of WBC, were positively associated with the risk of SO in our study. As an immune cell type, monocytes play a key role in inflammatory diseases (SahBandar et al., 2020). It has been demonstrated that monocytes are linked to the progression of CVD, and the level of monocytes can reflect the severity of CVD (Williams et al., 2021). In our study, the proportion of subjects who had hypertension, heart disease, and stroke in the SO group was higher than the proportion of similar subjects in the NSO group. CVD is also accompanied by the pro-inflammatory state and insulin resistance (Wu and Ballantyne, 2017), which may lead to a decrease in muscle mass and an increase in fat mass, and ultimately develop into SO (Hong and Choi, 2020).

Our study has the following strengths. First, this is the first cross-sectional study to investigate and explore the prevalence and risk factors of S and SO in older people over the age of 65 years in the Chongming District, which is a suburban area of Shanghai, China. Currently, most of the studies were performed in urban areas and they rarely focused on the suburban population of China. There are significant differences in the economic and health status between urban and suburban areas. Thus, investigating and exploring the prevalence and risk factors of S and SO in suburban areas can improve the awareness of S and SO among clinical staff and older people, which is essential for the timely prevention and treatment of sarcopenia and SO. Second, our study was done on a relatively large scale that studied two towns, which included seven communities (a total of 1,407 subjects aged ≥65 years). To date, there is only one study that has investigated the prevalence of S and SO in a community of Shanghai (a total of 631 subjects aged >65 years) (Du et al., 2019). Third, the risk factors involved in our study were comprehensive, not only including anthropometric characteristics, demographic characteristics, and chronic diseases, but also including CVD risk factors and PA level and lifestyle. Fourth, previous studies mostly focused on sarcopenia (including S and SO) or only SO, respectively. And our study focused on the prevalence and risk factors of S and SO, simultaneously. Fifth, blood indicators involved in our study are available and inexpensive. In suburban communities, the assessments of typical inflammation factors (such as hypersensitive C-reactive protein, interleukin-6 (IL-6), and tumor necrosis factor-α (TNF-α) cytokines) are unavailable and expensive. In our study, we selected blood indicators involved in routine community medical examinations (such as WBC, blood platelet, and lymphocyte). It is necessary to explore the relationship between the blood indicators and the prevalence of S and SO by selecting easily available and inexpensive blood indicators, and provide early screening and timely prevention and treatment of S and SO, eventually to improve the quality of life and reduce mortality among older people in suburban communities.

Our study also has several limitations. First, the assessments of nutrition status, sleep duration, and PA level in our study were measured using self-reported questionnaires, therefore they may produce recall bias. Second, we found that over 90% of older people in the Chongming District were at risk of malnutrition, and it may be related to the dietary structure among older people. In the future, we will perform more studies to explore the relationship between nutrition and sarcopenia or SO in the Chongming District.

## 5. Conclusion

To conclude, we found that the prevalence of S and SO was 9.74 and 9.95% among older people in the Chongming District, Shanghai. Additionally, the risk factors of S included lower BMI, lower HC, farming, higher HDL-C level, and a sleep duration <7 h, meanwhile aging, male gender, higher BMI, higher monocyte level, and a sleep duration >9 h were risk factors for SO. These results indicated that it is necessary to improve the awareness of S and SO, decrease the time of farming; meanwhile, it is necessary to increase PA level, keep appropriate sleep duration, and avoid other risk factors in life that prevent the occurrence of S and SO.

## Data availability statement

The original contributions presented in the study are included in the article/Supplementary material, further inquiries can be directed to the corresponding authors.

## Ethics statement

The studies involving human participants were reviewed and approved by the project of National Natural Science Foundation of China Mechanism of the Regulation of Skeletal Muscle Cell AMPK Pathway by Intestinal P.merdae in the Progression of Sarcopenia (No. 82102651); the Special Health Research Project of Shanghai Municipal Health Commission on the Health of Aging, Woman and Children, Exploration on the Screening and Rehabilitation Intervention Model for Sarcopenia among Community-dwelling older people in Chongming District under the Medical Union Model (No. 2020YJZX0137). The patients/participants provided their written informed consent to participate in this study. Written informed consent was obtained from the individual(s) for the publication of any potentially identifiable images or data included in this article.

## Author contributions

LL, XH, and YS participated in protocol design, investigation, methodology, formal analysis, and manuscript preparation. MZ participated in data curation. XW and NC participated in protocol design, resources, manuscript revision, and funding acquisition. All authors have read and approved the final manuscript.
