# Supplementary material for: Prevalence and risk factors of sarcopenia without obesity and sarcopenic obesity among Chinese community older people in suburban area of Shanghai: A cross-sectional study
Source: Front Aging Neurosci. 2022 Dec 20;14:1034542. doi: 10.3389/fnagi.2022.1034542 (PMC9807613; doi:10.3389/fnagi.2022.1034542)
Supplement: Supplementary file 1 [file Table_1.DOCX]

**Supplementary Table 1a. Clinical characteristics and differences between non-sarcopenic obesity group and sarcopenic obesity group**

| **Variables** | **Total**  **(n=1407)** | **Non-Sarcopenic obesity** | | | | **Sarcopenic obesity**  **(n=140)** | **p-value^1^** | **p-value^2^** |
| --- | --- | --- | --- | --- | --- | --- | --- | --- |
|  |  | **Total**  **(n=1276)** | **Normal**  **(n=388)** | **Sarcopenia**  **without obesity**  **(n=137)** | **Obesity**  **without sarcopenia**  **(n=742)** |  |  |  |
| **Age (y), mean±SD** | 71.91 (5.589) | 71.45 (5.273) | 70.25 (4.734) *** | 73.53 (6.501) *** | 71.70 (5.140) *** | 76.06 (6.592) | p<0.001 | p<0.001 |
| **Age (y), n (%)** |  |  |  |  |  |  | p<0.001 | p<0.001 |
| 65-69 y | 572 (40.7) | 547 (43.2) | 211 (54.4) ** | 44 (32.1) ** | 292 (39.4) ** | 25 (17.9) |  |  |
| 70-74 y | 436 (31.0) | 399 (31.5) | 106 (27.3) | 40 (29.2) | 253 (34.1) | 37 (26.4) |  |  |
| 75-79 y | 234 (16.6) | 200 (15.8) | 47 (12.1) ** | 28 (20.4) | 125 (16.8) | 34 (24.3) |  |  |
| ≥ 80 y | 165 (11.7) | 121 (9.6) | 24 (6.2) ** | 25 (18.2) | 72 (9.7) ** | 44 (31.4) |  |  |
| **Gender, n (%)** |  |  |  |  |  |  | p<0.001 | p<0.001 |
| Female | 826 (58.7) | 767 (60.5) | 284 (73.2) ** | 83 (60.6) ** | 400 (53.9) | 59 (42.1) |  |  |
| Male | 581 (41.3) | 500 (39.5) | 104 (26.8) ** | 54 (39.4) ** | 342 (46.1) | 81 (57.9) |  |  |
| **Anthropometric characteristics** | |  |  |  |  |  |  |  |
| BMI (kg/m^2^), mean±SD | 24.645 (3.316) | 24.795 (3.375) | 22.999 (2.471) | 19.930 (1.704) *** | 26.633 (2.508) *** | 23.282 (2.325) | p<0.001 | p<0.001 |
| BMI (kg/m^2^), n (%) |  |  |  |  |  |  | p<0.001 | p<0.001 |
| < 18.5 kg/m^2^ | 34 (2.4) | 32 (2.5) | 6 (1.5) | 26 (19.0) ** | /** | 2 (1.4) |  |  |
| 18.5 ≤ BMI ≤ 23.9 kg/m^2^ | 569 (40.4) | 474 (37.4) | 273 (70.4) | 110 (80.3) | 91 (12.3) ** | 95 (67.9) |  |  |
| ≥ 24.0 kg/m^2^ | 804 (57.1) | 761 (60.1) | 109 (28.1) | 1 (0.7) ** | 651 (87.7) ** | 43 (30.7) |  |  |
| WC (cm), mean±SD | 88.341 (9.590) | 88.551 (9.784) | 83.635 (8.183) ** | 77.385 (7.673) *** | 93.182 (7.785) *** | 86.450 (7.369) | p=0.002 | p<0.001 |
| HC (cm), mean±SD | 97.235 (6.533) | 97.445 (6.627) | 94.353 (5.249) | 89.906 (4.769) *** | 100.453 (5.647) *** | 95.338 (5.259) | p<0.001 | p<0.001 |
| PBF (%), mean±SD | 32.550 (6.760) | 32.288 (6.826) | 27.988 (5.604) *** | 25.912 (5.713) *** | 35.713 (5.317) | 34.922 (5.616) | p<0.001 | p<0.001 |
| **Demographic characteristics** |  |  |  |  |  |  |  |  |
| Farming, n (%) |  |  |  |  |  |  | p=0.908 | p=0.068 |
| No | 710 (50.5) | 640 (50.5) | 210 (54.1) | 56 (40.9) | 374 (50.4) | 70 (50.0) |  |  |
| Yes | 697 (49.5) | 627 (49.5) | 178 (45.9) | 81 (59.1) | 368 (49.6) | 70 (50.0) |  |  |
| Educational level, n (%) |  |  |  |  |  |  | p=0.144 | p=0.059 |
| Low | 731 (52.0) | 648 (51.1) | 195 (50.3) | 71 (51.8) | 382 (51.5) | 83 (59.3) |  |  |
| Moderate | 643 (45.7) | 590 (46.6) | 188 (48.5) | 66 (48.2) | 336 (45.3) | 53 (37.9) |  |  |
| High | 33 (2.3) | 29 (2.3) | 5 (1.3) | / | 24 (3.2) | 4 (2.9) |  |  |
| Smoking status, n (%) |  |  |  |  |  |  | p=0.001 | p<0.001 |
| Never smoking | 1009 (71.7) | 927 (73.2) | 312 (80.4) ** | 96 (70.1) | 519 (69.9) ** | 82 (58.6) |  |  |
| Current smoking | 225 (16.0) | 190 (15.0) | 50 (12.9) ** | 26 (19.0) | 114 (15.4) ** | 35 (25.0) |  |  |
| Former smoking | 173 (12.3) | 150 (11.8) | 26 (6.7) ** | 15 (10.9) | 109 (14.7) | 23 (16.4) |  |  |
| Drinking status, n (%) |  |  |  |  |  |  | p=0.113 | p<0.001 |
| Never drinking | 909 (64.6) | 825 (65.1) | 281 (72.4) ** | 100 (73.0) | 444 (59.8) | 84 (60.0) |  |  |
| Current drinking | 373 (26.5) | 336 (26.5) | 90 (23.2) | 24 (17.5) | 222 (29.9) | 37 (26.4) |  |  |
| Former drinking | 125 (8.9) | 106 (8.4) | 17 (4.4) ** | 13 (9.5) | 76 (10.2) | 19 (13.6) |  |  |
| Nutritional status, n (%) |  |  |  |  |  |  | p=0.420 | p<0.001 |
| Protein-calorie malnutrition | 16 (1.1) | 14 (1.1) | 2 (0.5) | 10 (7.3) | 2 (0.3) | 2 (1.4) |  |  |
| At risk of malnutrition | 1282 (91.1) | 1151 (90.8) | 371 (95.6) | 127 (92.7) | 653 (88.0) | 131 (93.6) |  |  |
| Adequate nutritional status | 109 (7.7) | 102 (8.1) | 15 (3.9) | /** | 87 (11.7) | 7 (5.0) |  |  |
| Depression status, n (%) |  |  |  |  |  |  | p=0.088 | p=0.069 |
| No (< 5) | 1281 (91.0) | 1159 (91.5) | 359 (92.5) | 119 (86.9) | 681 (91.8) | 122 (87.1) |  |  |
| Yes (≥ 5) | 126 (9.0) | 108 (8.5) | 29 (7.5) | 18 (13.1) | 61 (8.2) | 18 (12.9) |  |  |
| **Chronic diseases** |  |  |  |  |  |  |  |  |
| Number of comorbidities, n (%) |  |  |  |  |  |  | p=0.116 | p=0.003 |
| 0 | 137 (9.7) | 128 (10.1) | 44 (11.3) | 25 (18.2) ** | 59 (8.0) | 9 (6.4) |  |  |
| 1 | 624 (44.3) | 554 (43.7) | 161 (41.5) | 63 (46.0) | 330 (44.5) | 70 (50.0) |  |  |
| 2 | 454 (32.3) | 417 (32.9) | 125 (32.2) | 37 (27.0) | 255 (34.4) | 37 (26.4) |  |  |
| ≥ 3 | 192 (13.6) | 168 (13.3) | 58 (14.9) | 12 (8.8) | 98 (13.2) | 24 (17.1) |  |  |
| Hypertension |  |  |  |  |  |  | p=0.121 | p<0.001 |
| No | 281 (20.0) | 260 (20.5) | 86 (22.2) | 45 (32.8) | 129 (17.4) | 21 (15.0) |  |  |
| Yes | 1126 (80.0) | 1007 (79.5) | 302 (77.8) | 92 (67.2) | 613 (82.6) | 119 (85.0) |  |  |
| Dyslipidaemia |  |  |  |  |  |  | p=0.402 | p=0.102 |
| No | 961 (68.3) | 861 (68.0) | 247 (63.7) | 100 (73.0) | 514 (69.3) | 100 (71.4) |  |  |
| Yes | 446 (31.7) | 406 (32.0) | 141 (36.3) | 37 (27.0) | 228 (30.7) | 40 (28.6) |  |  |
| Diabetes |  |  |  |  |  |  | p=0.890 | p=0.187 |
| No | 1052 (74.8) | 948 (74.8) | 287 (74.0) | 113 (82.5) | 548 (73.9) | 104 (74.3) |  |  |
| Yes | 355 (25.2) | 319 (25.2) | 101 (26.0) | 24 (17.5) | 194 (26.1) | 36 (25.7) |  |  |
| Heart disease |  |  |  |  |  |  | p=0.704 | p=0.912 |
| No | 1317 (93.6) | 1187 (93.7) | 366 (94.3) | 128 (93.4) | 693 (93.4) | 130 (92.9) |  |  |
| Yes | 90 (6.4) | 80 (6.3) | 22 (5.7) | 9 (6.6) | 49 (6.6) | 10 (7.1) |  |  |
| Stroke |  |  |  |  |  |  | p=0.648 | p=0.925 |
| No | 1281 (91.0) | 1155 (91.2) | 356 (91.8) | 125 (91.2) | 674 (90.8) | 126 (90.0) |  |  |
| Yes | 126 (9.0) | 112 (8.8) | 32 (8.2) | 12 (8.8) | 68 (9.2) | 14 (10.0) |  |  |
| BMI: body mass index; WC: waist circumference; HC: hip circumference; PBF: percent of body fat; p-value^1^: difference between non-sarcopenic obesity group and sarcopenic obesity group; p-value^2^: difference between normal, sarcopenia without obesity, obesity without sarcopenia and sarcopenic obesity group; *: significantly difference between sarcopenia without obesity group and sarcopenic obesity group; p<0.1*, p<0.05**, p<0.001*** | | | | | | | | |

**Supplementary Table 1b. Cardiovascular disease risk factors characteristics and differences between non-sarcopenic obesity group and sarcopenic obesity group**

| **Variables** | **Total**  **(n=1407)** | **Non-Sarcopenic obesity** | | | | **Sarcopenic obesity**  **(n=140)** | **p-value^1^** | **p-value^2^** |
| --- | --- | --- | --- | --- | --- | --- | --- | --- |
|  |  | **Total**  **(n=1276)** | **Normal**  **(n=388)** | **Sarcopenia**  **without obesity**  **(n=137)** | **Obesity**  **without sarcopenia**  **(n=742)** |  |  |  |
| **Inflammation factors, mean±SD** | | | | | | | | |
| WBC (10^9/L) | 6.163 (1.503) | 6.149 (1.505) | 6.001 (1.475) | 6.004 (1.899) | 6.252 (1.430) | 6.296 (1.486) | p=0.273 | p=0.024 |
| Blood platelet (10^9/L) | 200.233 (53.889) | 201.557 (52.574) | 203.008 (55.119) ** | 198.387 (52.978) | 201.385 (51.162) ** | 188.245 (63.556) | p=0.018 | p=0.095 |
| Lymphocyte (10^9/L) | 2.163 (0.758) | 2.172 (0.760) | 2.127 (0.699) | 2.022 (0.907) | 2.223 (0.757) | 2.080 (0.740) | p=0.176 | p=0.008 |
| Monocytes (10^9/L) | 0.429 (0.142) | 0.425 (0.129) | 0.416 (0.126) ** | 0.399 (0.129) ** | 0.434 (0.130) | 0.462 (0.225) | p=0.055 | p=0.002 |
| Neutrophils (10^9/L) | 3.396 (1.155) | 3.377 (1.147) | 3.313 (1.165) | 3.396 (1.304) | 3.407 (1.106) | 3.571 (1.218) | p=0.059 | p=0.152 |
| Eosinophils (10^9/L) | 0.179 (0.146) | 0.179 (0.164) | 0.155 (0.105) | 0.196 (0.223) | 0.188 (0.174) | 0.181 (0.146) | p=0.891 | p<0.001 |
| PLR | 103.514 (87.145) | 104.002 (90.751) | 110.409 (151.371) | 111.121 (47.220) | 99.337 (40.521) | 99.099 (42.258) | p=0.528 | p=0.136 |
| LMR | 5.441 (2.404) | 5.488 (2.414) | 5.479 (2.245) | 5.255 (1.890) | 5.535 (2.580) * | 5.015 (2.272) | p=0.027 | p=0.091 |
| NLR | 1.774 (1.169) | 1.757 (1.187) | 1.776 (1.569) | 1.879 (0.931) | 1.725 (0.979) | 1.927 (0.988) | p=0.103 | p=0.187 |
| **Blood lipid factors, mean±SD** | | | | | | | | |
| TCHOL (mmol/L) | 5.125 (1.031) | 5.134 (1.033) | 5.202 (1.092) | 5.192 (0.979) | 5.087 (1.009) | 5.044 (1.020) | p=0.329 | p=0.207 |
| TG (mmol/L) | 1.718 (1.358) | 1.744 (1.409) | 1.857 (1.898) ** | 1.401 (1.007) | 1.748 (1.138) | 1.480 (0.711) | p<0.001 | p<0.001 |
| LDL-C (mmol/L) | 2.956 (0.837) | 2.967 (0.835) | 2.980 (0.888) | 2.866 (0.804) | 2.979 (0.811) | 2.859 (0.857) | p=0.147 | p=0.228 |
| HDL-C (mmol/L) | 2.010 (0.491) | 2.005 (0.490) | 2.054 (0.508) | 2.304 (0.568) *** | 1.925 (0.439) ** | 2.050 (0.500) | p=0.309 | p<0.001 |
| WBC: white blood cell; PLR: platelet to lymphocytes; LMR: lymphocytes to monocytes; NLR: neutrophils to lymphocytes; TCHOL: total cholesterol; TG: triglycerides; LDL-C: low-density lipoprotein cholesterol; HDL-C: high-density lipoprotein cholesterol; p-value^1^: difference between non-sarcopenic obesity group and sarcopenic obesity group; p-value^2^: difference between normal, sarcopenia without obesity, obesity without sarcopenia and sarcopenic obesity group; *: significantly difference between sarcopenia without obesity group and sarcopenic obesity group; p<0.1*, p<0.05**, p<0.001*** | | | | | | | | |

**Supplementary Table 1c. Physical activity and lifestyle characteristics and differences between non-sarcopenic obesity group and sarcopenic obesity group**

| **Variables** | **Total**  **(n=1407)** | **Non-Sarcopenic obesity** | | | | **Sarcopenic obesity**  **(n=140)** | **p-value^1^** | **p-value^2^** |
| --- | --- | --- | --- | --- | --- | --- | --- | --- |
|  |  | **Total**  **(n=1276)** | **Normal**  **(n=388)** | **Sarcopenia without obesity**  **(n=137)** | **Obesity**  **without sarcopenia**  **(n=742)** |  |  |  |
| **PA level, n (%)** |  |  |  |  |  |  | p=0.465 | p=0.605 |
| Low level | 635 (45.1) | 570 (45.0) | 172 (44.3) | 66 (48.2) | 332 (44.7) | 65 (46.4) |  |  |
| Moderate level | 584 (41.5) | 523 (41.3) | 157 (40.5) | 50 (36.5) | 316 (42.6) | 61 (43.6) |  |  |
| High level | 188 (13.4) | 174 (13.7) | 59 (15.2) | 21 (15.3) | 94 (12.7) | 14 (10.0) |  |  |
| **Lifestyle** |  |  |  |  |  |  |  |  |
| Exercise habits, n (%) |  |  |  |  |  |  | p=0.306 | p=0.727 |
| Yes | 157 (11.2) | 145 (11.4) | 47 (12.1) | 15 (10.9) | 83 (11.2) | 12 (8.6) |  |  |
| No | 1250 (88.8) | 1122 (88.6) | 341 (87.9) | 122 (89.1) | 659 (88.8) | 128 (91.4) |  |  |
| Activity time (min/w), mean±SD | | | | | | | | |
| Sitting time | 1194.24 (724.143) | 1184.66 (737.148) | 1042.33 (663.273) ** | 1243.14 (705.768) | 1248.28 (769.353) | 1281.00 (588.766) | p=0.075 | p<0.001 |
| Housework time | 991.94 (871.297) | 1017.22 (873.673) | 1104.97 (948.104) *** | 971.72 (912.298) | 979.74 (822.231) ** | 763.14 (817.497) | p=0.001 | p=0.001 |
| Sleep duration (h/d), n (%) | |  |  |  |  |  | p=0.010 | p=0.003 |
| < 7 h | 455 (32.3) | 413 (32.6) | 122 (31.4) | 53 (38.7) | 238 (32.1) | 42 (30.0) |  |  |
| 7-9 h | 726 (51.6) | 663 (52.3) | 216 (55.7) | 55 (40.1) | 392 (52.8) | 63 (45.0) |  |  |
| > 9 h | 226 (16.1) | 191 (15.1) | 50 (12.9) ** | 29 (21.2) | 112 (15.1) ** | 35 (25.0) |  |  |
| PA: physical activity; p-value^1^: difference between non-sarcopenic obesity group and sarcopenic obesity group; p-value^2^: difference between normal, sarcopenia without obesity, obesity without sarcopenia and sarcopenic obesity group; *: significantly difference between sarcopenia without obesity group and sarcopenic obesity group; p<0.1*, p<0.05**, p<0.001*** | | | | | | | | |

**Supplementary Table 2. Univariate and multiple logistic regression analysis of sarcopenia without obesity and sarcopenic obesity**

| **Factors** | **Sarcopenia without obesity** | | **Sarcopenic obesity** | |
| --- | --- | --- | --- | --- |
|  | **Univariate logistic**  **regression analysis** | **Multiple logistic**  **regression analysis** | **Univariate logistic**  **regression analysis** | **Multiple logistic**  **regression analysis** |
| **Age (y), n (%)** |  |  |  |  |
| 65-69 y | **1 (Reference) (p=0.024)** | **1 (Reference) (p=0.353)** | **1 (Reference) (p<0.001)** | **1 (Reference) (p<0.001)** |
| 70-74 y | 1.212 (0.775-1.896) (p=0.400) | (p=0.768) | 2.029 (1.202-3.425) (p=0.008) | 1.923 (1.122-3.295) (p=0.017) |
| 75-79 y | 1.631 (0.989-2.690) (p=0.055) | (p=0.362) | 3.720 (2.165-6.390) (p<0.001) | 3.185 (1.816-5.585) (p<0.001) |
| ≥ 80 y | 2.143 (1.268-3.623) (p=0.004) | (p=0.345) | 7.956 (4.688-13.502) (p<0.001) | 7.192 (4.133-12.513) (p<0.001) |
| **Gender, n (%)** |  |  |  |  |
| Female | **1 (Reference)** | --- | **1 (Reference)** | **1 (Reference)** |
| Male | 0.917 (0.640-1.315) (p=0.639) | --- | 2.106 (1.478-3.000) (p<0.001) | 1.981 (1.351-2.904) (p<0.001) |
| **Anthropometric characteristics** | |  |  |  |
| BMI (kg/m^2^), n (%) |  |  |  |  |
| < 18.5 kg/m^2^ | **1 (Reference) (p<0.001)** | **1 (Reference) (p<0.001)** | **1 (Reference) (p<0.001)** | **1 (Reference) (p<0.001)** |
| 18.5 ≤ BMI ≤ 23.9 kg/m^2^ | 0.074 (0.033-0.167) (p<0.001) | 0.136 (0.054-0.340) (p<0.001) | 3.207 (0.756-13.609) (p=0.114) | 4.865 (1.089-21.736) (p=0.038) |
| ≥ 24.0 kg/m^2^ | 0.000 (0.000-0.003) (p<0.001) | 0.002 (0.000-0.019) (p<0.001) | 0.904 (0.210-3.898) (p=0.892) | 1.255 (0.277-5.679) (p=0.768) |
| WC (cm), mean±SD | 0.845 (0.823-0.868) (p<0.001) | (p=0.115) | 0.977 (0.959-0.995) (p=0.014) | (p=0.700) |
| HC (cm), mean±SD | 0.762 (0.729-0.796) (p<0.001) | 0.858 (0.816-0.903) (p<0.001) | 0.950 (0.924-0.977) (p<0.001) | (p=0.645) |
| **Demographic characteristics** |  |  |  |  |
| Farming, n (%) |  |  |  |  |
| No | **1 (Reference)** | **1 (Reference)** | **1 (Reference)** | --- |
| Yes | 1.536 (1.074-2.197) (p=0.019) | 1.632 (1.053-2.530) (p=0.028) | 1.021 (0.720-1.447) (p=0.908) | --- |
| Educational level, n (%) |  |  |  |  |
| Low | **1 (Reference) (p=0.944)** | --- | **1 (Reference) (p=0.146)** | --- |
| Moderate | 1.063 (0.747-1.514) (p=0.733) | --- | 0.701 (0.488-1.007) (p=0.055) | --- |
| High | 0.000 (0.000-) (p=0.998) | --- | 1.077 (0.369-3.140) (p=0.892) | --- |
| Smoking status, n (%) |  |  |  |  |
| Never smoking | **1 (Reference) (p=0.570)** | --- | **1 (Reference) (p=0.001)** | **1 (Reference) (p=0.382)** |
| Current smoking | 1.243 (0.785-1.968) (p=0.354) | --- | 2.082 (1.361-3.187) (p=0.001) | (p=0.260) |
| Former smoking | 0.903 (0.511-1.596) (p=0.725) | --- | 1.733 (1.058-2.840) (p=0.029) | (p=0.879) |
| Drinking status, n (%) |  |  |  |  |
| Never drinking | **1 (Reference) (p=0.045)** | **1 (Reference) (p=0.204)** | **1 (Reference) (p=0.119)** | --- |
| Current drinking | 0.556 (0.350-0.884) (p=0.013) | (p=0.108) | 1.082 (0.720-1.625) (p=0.706) | --- |
| Former drinking | 0.939 (0.510-1.729) (p=0.840) | (p=0.325) | 1.760 (1.029-3.013) (p=0.039) | --- |
| Nutritional status, n (%) |  |  |  |  |
| Protein-calorie malnutrition | **1 (Reference) (p<0.001)** | **1 (Reference) (p=0.080)** | **1 (Reference) (p=0.428)** | --- |
| At risk of malnutrition | 0.066 (0.024-0.185) (p<0.001) | 0.167 (0.035-0.795) (p=0.025) | 0.797 (0.179-3.544) (p=0.765) | --- |
| Adequate nutritional status | 0.000 (0.000-) (p=0.995) | 0.000 (0.000-) (p=0.995) | 0.480 (0.091-2.546) (p=0.389) | --- |
| Depression status, n (%) |  |  |  |  |
| No (< 5) | **1 (Reference)** | **1 (Reference)** | **1 (Reference)** | **1 (Reference)** |
| Yes (≥ 5) | 1.627 (0.955-2.774) (p=0.074) | (p=0.300) | 1.583 (0.929-2.697) (p=0.091) | (p=0.238) |
| **Chronic diseases, n (%)** |  |  |  |  |
| Number of comorbidities |  |  |  |  |
| 0 | **1 (Reference) (p=0.002)** | **1 (Reference) (p=0.233)** | **1 (Reference) (p=0.121)** | --- |
| 1 | 0.503 (0.303-0.834) (p=0.008) | (p=0.133) | 1.797 (0.875-3.693) (p=0.111) | --- |
| 2 | 0.398 (0.230-0.688) (p=0.001) | (p=0.244) | 1.262 (0.593-2.685) (p=0.546) | --- |
| ≥ 3 | 0.299 (0.144-0.618) (p=0.001) | (p=0.472) | 2.032 (0.913-4.521) (p=0.082) | --- |
| Hypertension |  |  |  |  |
| No | **1 (Reference)** | **1 (Reference)** | **1 (Reference)** | --- |
| Yes | 0.467 (0.318-0.685) (p<0.001) | (p=0.061) | 1.463 (0.902-2.373) (p=0.123) | --- |
| Dyslipidaemia |  |  |  |  |
| No | **1 (Reference)** | --- | **1 (Reference)** | --- |
| Yes | 0.779 (0.525-1.156) (p=0.215) | --- | 0.848 (0.577-1.247) (p=0.402) | --- |
| Diabetes |  |  |  |  |
| No | **1 (Reference)** | **1 (Reference)** | **1 (Reference)** | --- |
| Yes | 0.603 (0.381-0.953) (p=0.030) | (p=0.840) | 1.029 (0.690-1.534) (p=0.890) | --- |
| Heart disease |  |  |  |  |
| No | **1 (Reference)** | --- | **1 (Reference)** | --- |
| Yes | 1.032 (0.506-2.104) (p=0.931) | --- | 1.141 (0.577-2.257) (p=0.704) | --- |
| Stroke |  |  |  |  |
| No | **1 (Reference)** | --- | **1 (Reference)** | --- |
| Yes | 0.973 (0.522-1.815) (p=0.933) | --- | 1.146 (0.638-2.057) (p=0.648) | --- |
| **CVD risk factors, mean±SD** |  |  |  |  |
| Inflammation factors |  |  |  |  |
| WBC (10^9/L) | 0.922 (0.816-1.042) (p=0.192) | --- | 1.065 (0.952-1.191) (p=0.273) | --- |
| Blood platelet (10^9/L) | 0.999 (0.996-1.003) (p=0.673) | --- | 0.995 (0.992-0.999) (p=0.006) | (p=0.192) |
| Lymphocyte (10^9/L) | 0.734 (0.564-0.955) (p=0.021) | (p=0.398) | 0.842 (0.656-1.079) (p=0.174) | --- |
| Monocytes (10^9/L) | 0.146 (0.035-0.605) (p=0.008) | (p=0.370) | 4.565 (1.522-13.692) (p=0.007) | 4.203 (1.340-13.181) (p=0.014) |
| Neutrophils (10^9/L) | 1.000 (0.859-1.165) (p=0.999) | --- | 1.147 (0.995-1.323) (p=0.059) | (p=0.649) |
| Eosinophils (10^9/L) | 1.752 (0.720-4.262) (p=0.216) | --- | 1.076 (0.378-3.064) (p=0.891) | --- |
| PLR | 1.001 (0.999-1.002) (p=0.333) | --- | 0.998 (0.994-1.003) (p=0.461) | --- |
| LMR | 0.962 (0.888-1.042) (p=0.341) | --- | 0.904 (0.828-0.988) (p=0.025) | (p=0.597) |
| NLR | 1.066 (0.948-1.197) (p=0.286) | --- | 1.091 (0.974-1.221) (p=0.131) | --- |
| Blood lipid factors |  |  |  |  |
| TCHOL (mmol/L) | 1.072 (0.905-1.270) (p=0.422) | --- | 0.918 (0.774-1.090) (p=0.329) | --- |
| TG (mmol/L) | 0.716 (0.577-0.889) (p=0.002) | (p=0.754) | 0.805 (0.665-0.974) (p=0.026) | (p=0.477) |
| LDL-C (mmol/L) | 0.866 (0.699-1.072) (p=0.186) | **---** | 0.855 (0.692-1.057) (p=0.147) | --- |
| HDL-C (mmol/L) | 3.456 (2.455-4.864) (p<0.001) | 2.235 (1.484-3.367) (p<0.001) | 1.198 (0.846-1.698) (p=0.309) | --- |
| **Physical activity and lifestyle** | |  |  |  |
| PA level, n (%) |  |  |  |  |
| Low level | **1 (Reference) (p=0.435)** | --- | **1 (Reference) (p=0.468)** | --- |
| Moderate level | 0.807 (0.549-1.187) (p=0.277) | --- | 1.023 (0.707-1.479) (p=0.905) | --- |
| High level | 1.084 (0.644-1.824) (p=0.761) | --- | 0.706 (0.386-1.288) (p=0.256) | --- |
| Lifestyle |  |  |  |  |
| Exercise habits, n (%) |  |  |  |  |
| Yes | **1 (Reference)** | --- | **1 (Reference)** | --- |
| No | 1.024 (0.583-1.800) (p=0.935) | --- | 1.378 (0.744-2.554) (p=0.307) | --- |
| Activity time (min/w), mean±SD | |  |  |  |
| Sitting time | 1.000 (1.000-1.000) (p=0.405) | --- | 1.000 (1.000-1.000) (p=0.136) | --- |
| Housework time | 1.000 (1.000-1.000) (p=0.775) | --- | 1.000 (0.999-1.000) (p=0.001) | (p=0.100) |
| Sleep duration (h/d), n (%) |  |  |  |  |
| < 7 h | **1 (Reference) (p=0.018)** | **1 (Reference) (p=0.013)** | **1 (Reference) (p=0.011)** | **1 (Reference) (p=0.023)** |
| 7-9 h | 0.622 (0.418-0.925) (p=0.019) | 0.561 (0.346-0.909) (p=0.019) | 0.934 (0.621-1.407) (p=0.745) | 1.006 (0.650-1.559) (p=0.978) |
| > 9 h | 1.117 (0.688-1.811) (p=0.655) | 1.283 (0.676-2.433) (p=0.446) | 1.802 (1.115-2.913) (p=0.016) | 1.881 (1.117-3.166) (p=0.017) |
| BMI: body mass index; WC: waist circumference; HC: hip circumference; PBF: percent of body fat; CVD: cardiovascular disease; WBC: white blood cell; PLR: platelet to lymphocytes; LMR: lymphocytes to monocytes; NLR: neutrophils to lymphocytes; TCHOL: total cholesterol; TG: triglycerides; LDL-C: low-density lipoprotein cholesterol; HDL-C: high-density lipoprotein cholesterol; PA: physical activity | | | | |
